# Molecular Docking Study of the C-10 Massoia Lactone Compound as an Antimicrobial and Antibiofilm Agent against *Candida tropicalis*

**DOI:** 10.1155/2023/6697124

**Published:** 2023-09-19

**Authors:** Hasyrul Hamzah, Titik Nuryastuti, Widya Rahmah, Lutfi Chabib, Eka Siswanto Syamsul, Dwi Lestari, Asriullah Jabbar, Sylvia Utami Tunjung Pratiwi

**Affiliations:** ^1^Faculty of Medicine, Public Health and Nursing, Universitas Gadjah Mada, Yogyakarta 55281, Indonesia; ^2^Faculty of Pharmacy, Universitas Muhammadiyah Kalimantan Timur, Samarinda, Kalimantan Timur 75124, Indonesia; ^3^Indonesian Biofilm Research Collaboration Centre (IBRCC), Farmako Street, Sekip Utara, Yogyakarta 55281, Indonesia; ^4^Department of Microbiology, Faculty of Medicine, Public Health and Nursing, Universitas Gadjah Mada, Farmako Street, North Sekip, Yogyakarta 55281, Indonesia; ^5^Department of Pharmacy, Islamic University of Indonesia, Yogyakarta, Indonesia; ^6^Department of Pharmacy, Sekolah Tinggi Ilmu Kesehatan Samarinda, Samarinda, East Borneo, Indonesia; ^7^Department of Pharmacy, Faculty of Pharmacy, Haluoleo University, Kendari 93232, Indonesia; ^8^Faculty of Pharmacy, Universitas Gadjah Mada, North Sekip, Yogyakarta 55281, Indonesia

## Abstract

Antimicrobial resistance is now considered a global health problem because it reduces the effectiveness of antimicrobial drugs. According to the World Health Organization (WHO), the highest mortality rate is associated with infections caused by multidrug-resistant microorganisms, with approximately 700,000 deaths worldwide each year. The aim of this study was to determine the potential of C-10 massoia lactone to inhibit the growth of fungi and *C. tropicalis* biofilm, and molecular docking studies were performed to determine the nature of the inhibition. The study was conducted using the microdilution method for antifungal and antibiofilm testing and designed with a molecular docking approach. Furthermore, an analysis using the scanning electron microscope (SEM) was performed to evaluate the mechanism of effect. The results obtained showed that C-10 massoia lactone can inhibit the growth of fungi by 84.21% w/v. Meanwhile, the growth of *C. tropicalis* biofilm in the intermediate phase was 80.23% w/v and in the mature phase was 74.23% w/v. SEM results showed that C-10 massoia lactone damaged the EPS matrix of *C. tropicalis* so that hyphal formation was hindered due to damage to fungal cells, resulting in a decrease in attachment, density, and lysis of *C. tropicalis* fungal cells. Based on molecular docking tests, C-10 massoia lactone was able to inhibit biofilm formation without affecting microbial growth, while docking C-10 massoia lactone showed a significant binding and has the potential as an antifungal agent. In conclusion, the C-10 massoia lactone compound has the potential as an antibiofilm against *C. tropicalis*, so it can become a new antibiofilm agent.

## 1. Introduction

Biofilm is one of the problems in the treatment of human infections, making it a health problem throughout the world, especially for patients suffering from immune system disorders such as cancer, organ transplants, urinary tract disorders, and malnutrition [1]. On the surface of hosts and inanimate objects such as tissues, blood vessels/heart valves, and wounds on the body or medical devices, biofilms can grow simply by adhering to the surface to form a group of microorganisms to form biofilms [2–4].

Biofilm bacteria are 1000 times more resistant to antibiotics than planktonic cells, which do not form biofilms [5]. There are few available antibiotics that can effectively combat biofilm infections, resulting in very high levels of drug resistance. To date, there has been an increase in biofilm-associated infections [6]. By forming biofilms, bacteria are able to protect themselves from antibiotics and disinfectants; therefore, antibiotic resistance often occurs in biofilms [2–4]. This is because biofilms contain extracellular polymeric substances (EPS) that envelop or wrap a collection of microorganisms that gather into colonies, making it difficult for antibiotics to penetrate the EPS to attack these microorganisms [7]. In addition, phenotypic and genotypic changes in gene expression are another cause of antibiotic resistance in biofilms [8].

This makes biofilm bacteria very resistant to antibiotic treatment and immune responses. Although it is known that antibiotic treatment is currently the most important and effective way to control microbial infections, it is almost impossible for antibiotic treatment to eradicate biofilm infection. Biofilm development via bacterial adhesion to human tissues and medical devices is an important virulence factor associated with increased antibiotic resistance, decreased phagocytosis, and overall survival of microorganisms [9–11].

The management of infections caused by biofilms is very important because infections caused by biofilms can affect the morbidity and mortality of a person and can cause death if not properly treated [8, 12]. It is known that biofilm infections cause the death of 7500 people per year [13]. Biofilms also cause economic losses of up to $11 billion in the United States [5, 14]. According to the World Health Organization (WHO), infections are the second leading cause of death in the world, and according to the US National Institutes of Health, infections caused by biofilms account for over 60% of all infections [15]. Furthermore, 80% of infections are caused by biofilms [16]. In several countries, the prevalence of biofilm infection in UTI cases is 46% in Thailand, 72% in India, and 95% in Iran [17].

In vitro and in vivo experiments show that the minimum inhibitory concentration (MIC) and minimum bactericidal concentration (MBC) for biofilm bacterial cells are usually much higher (about 10–1000 times) than those for planktonic bacterial cells [18–21].

Therefore, due to the increasing antibiotic resistance of clinically relevant bacteria, there is an urgent need to develop new classes of antibiotics that are not affected by preexisting resistance mechanisms in the bacterial population [22, 23]. There is also a need for new anti-infectives that work through new mechanisms of action [24]. Plants are a good source of natural materials to find bioactive compounds [25, 26]. The study of alternative mechanisms of antibiotic resistance can open new avenues for the development of antibiotic resistance [24], and natural products can become an important reservoir of antibiotics to overcome mechanisms of antibiotic resistance [27, 28].

C-10 massoia lactone is a compound from the masoyi plant (*Cryptocarya massoy*) which has many health benefits such as antibiofilm [29], antibacterial [30], antifungal [29–31], antioxidant, antidiabetic, and anticytotoxic [32], and immolator [33]. Other microbes inhibited by the C-10 massoia lactone compound included *Pseudomonas aeruginosa*, *Candida albicans*, and *Staphylococcus aureus* [34], *Actinomyces viscosus*, *Streptococcus mutans*, *Streptococcus sanguinis*, and *Lactobacillus acidophilus* [35], and *Escherichia coli* and *Pseudomonas aeruginosa* [29].

Therefore, the aim of this study was to investigate the inhibition of the antibiofilm activity of the C-10 massoia lactone and to identify the targets of its inhibitory effect on biofilms using molecular docking.

## 2. Materials and Methods

### 2.1. Instruments

Instruments used in this study were as follows: autoclave (Sakura, Japan), Laminar Discuss Stream, 24-well flat-bottomed polystyrene microplates, 96-well flat-bottomed polystyrene microplates (Iwaki, Japan), multichannel micropipette (Socorex, Switzerland), micropipette (Gilson, France), microtiter plate reader (Optic Ivymen Framework 2100-C, Spain), hatchery (IF-2B) (Sakura, Japan), spectrophotometer (Genesys 10 UV Filtering, 335903) (Thermo Logical Spectronic, USA), and balance (AB204-5, Switzerland).

### 2.2. Materials

Materials used in this study were as follows: masoyi plant (novel C-10 massoia lactone), *C. tropicalis* isolates forming a biofilm standard (*C. tropicalis* JFM 1541) from the collection of the Microbiology Laboratory of the UGM Faculty of Medicine, sterile distilled water, RPMI media, SDA (Sabouraud dextrose agar) media, PBS (phosphate buffer saline) solution, DMSO 1%, nystatin, NaCl, McFarland standard 0.5, 1% crystal violet, disposable mask, and gloves.

### 2.3. Fungal Preparation for Testing

The test isolate used in this study was *C. tropicalis* JFM 1541. First, several fungal colonies on SDA media were inoculated into 15 mL of YPD medium, which was then incubated at room temperature in a shaker for 24 hours. The fungal culture was then centrifuged at 3000 rpm for 15 minutes and washed with PBS. The pellets obtained were then resuspended in RPMI medium, and a *C. tropicalis* 1 × 10^8^ CFU/mL suspension was prepared using 5 mL of RPMI medium (equivalent to McFarland standard 0.5). Then, 1 mL of the suspension was added to 9 mL of RPMI medium to obtain a 1 × 10^7^ CFU/mL *C. tropicalis* colony suspension as a stock solution. To obtain a 1 × 10^6^ CFU/mL *C. tropicalis* suspension, the working solution was prepared at a 1 : 10 dilution [36].

### 2.4. Antifungal Testing Using the Microdilution Method

Antifungal testing was performed using the microdilution method, where samples were pure isolates with several concentration series (0.125%–1% w/v). Nystatin 1% w/v was used as a positive control for comparison with the study results. This test was performed on 96-well flat-bottomed polystyrene microtiter plates, with the sample placed in each microtiter plate well containing RPMI media. The samples were then incubated at 37°C for 72 hours, after which the absorbance was read using a microplate reader at a wavelength of 595 nm.

### 2.5. Determination of Minimum Biofilm Inhibitory Concentration (MBIC) Using the Microbroth Dilution Method

The novel C-10 massoia lactone was tested on the biofilm strain *C. tropicalis* JFM 1541. The biofilm was inoculated into 96-well flat-bottomed polystyrene microplates. Each well received 200 *μ*L of *C. tropicalis* suspension (10^6^ CFU/mL) and was then incubated at ± 37°C for 90 minutes [37]. After incubation, the plates were washed with PBS. Media containing pure isolate at a concentration (0.125%–1% w/v) of 200 *μ*L was then added to each well. Media containing 1% DMSO was used as a solvent control, and microbial suspension was used as a negative control. The antifungal drug nystatin was added to the microbial suspension at 1% w/v as a positive control and media with no added microbes as a control medium [38]. The plates were then incubated at 37°C for 24 hours to form biofilms in the intermediate phase and for 48 hours in the maturation phase [39, 40]. The plates were then washed with PBS. 125 *μ*L of 1% crystal violet solution was added to each well and incubated for 15 minutes at room temperature. After incubation, the microtiter plate was washed with PBS, and 200 *μ*L of 96% ethanol was added to each well to dissolve the biofilm. Optical density (OD) readings were performed using a microplate reader at a wavelength of 595 nm [39–42].

### 2.6. Scanning Electron Microscopy (SEM) Testing

For the scanning electron microscopy (SEM) test, 24-well flat-bottomed polystyrene microplates containing the test suspension were treated in the same way as for the biofilm test. The microplates were then incubated at 37°C for 24–48 hours. After incubation, the wells containing the test sample on the microplate were carefully rinsed three times with sterile distilled water. Then, the wells containing the sample were fixed with cacodylate internal glutaraldehyde 0.5% (v/v) for ±24 hours at the cell passing point without changing the cell structure to be observed. The microplate was then hydrated with methanol for 30 minutes to reduce the water content in the wells so that the results could be clearly seen. Scanning electron microscopy was used to examine the samples at a voltage of 10 kV [42, 43].

### 2.7. Molecular Docking

The possible pathway for the binding of phytochemicals found in the massoia lactone compound to the protein 3HEM (mycolic acid cyclopropane synthase CmaA2 in complex with dioctylamine) is a protein target from the bacteria/EPS biofilm matrix, which was modelled using molecular docking. The three-dimensional structure of the 3HEM matrix was obtained from the RCSB protein database. The data were then prepared by removing crystallographic water and crystallised ligands. Molecular docking simulations were performed using AutoDock Vina (Vina) [44]. The intermolecular interactions of the protein-ligand complex are inferred using the PoseView accessible via Protein PDB. Optimisation is performed by adding hydrogen atoms and determining the grid box parameters. The addition of a hydrogen atom is essential for ligand and receptor interactions. The hydrogen atom, which is automatically added by the program, is polar as it is involved in hydrogen bonding. Determining the grid box parameter produces a three-dimensional map of protein interactions with each type of atom found in the ligand [45].

### 2.8. Statistical Method

The statistical analysis of the research results was carried out using the ANOVA normality test, which was performed using the Shapiro–Wilk method. The normality level of the test is *P* < 0.05, and the data were analysed using the Statistical Package for the Social Sciences (SPSS).

## 3. Result and Discussion

Microdilution is an antifungal and antibiofilm testing method that uses the same principle as the liquid dilution method but with small amounts of compounds, media, fungi, and bacteria using a flat-bottomed polystyrene microtiter plate with 96 wells. With research results that are more sensitive and effective in quality, use small samples, and are efficient, this method is preferred for further research because it can test many samples quickly.

### 3.1. The Results of the Antifungal Test Using the Microdilution Method

**Figure 1:**
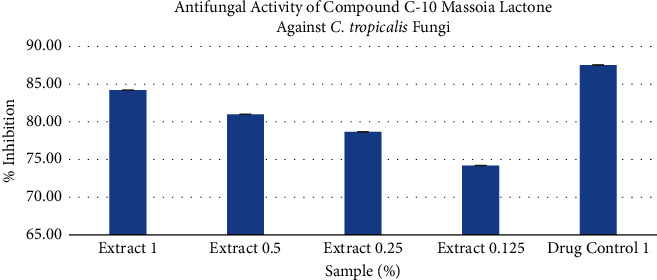
Antifungal activity of compound C-10 massoia lactone against *C. tropicalis* fungus.

Based on the research results, the C-10 massoia lactone compound provided an antifungal inhibitory activity against *C. tropicalis* of 84.21% ± 0.01. Although not as great as the positive control which had an inhibitory activity of 87.55% ± 0.01, the C-10 massoia lactone compound provided almost the same inhibitory activity as the positive control nystatin. In addition, at the lowest concentration (0.125%), it could still give an inhibitory activity of 74.21% ± 0.01 against *C. tropicalis* (Figure 1 and Table 1).

According to Zhang et al. [31], the C-10 massoia lactone compound has antifungal activity against *Fusarium graminearum* spores, where C-10 massoia lactone inhibits the growth of *Fusarium graminearum* by interfering with the formation of pores in the fungal cell membrane, resulting in increase in intracellular ROS levels, decrease in ergosterol content, and leakage of intracellular components within the fungus, leading to cellular necrosis and fungal cell death.

In addition, according to Zoccolotti et al. [46], masoyi extract reduced *Candida albicans* cell metabolism from fungal growth to the formation of *Candida albicans* biofilms, where masoyi extract reduced cell viability, which was better than the control group (nystatin) in its cytotoxicity test.

### 3.2. Determination of Minimum Biofilm Inhibitory Concentration (MBIC_50_) Using the Microbroth Dilution Method

Biofilms in healthcare are one of the problems in dealing with infections associated with human diseases, one of which is nosocomial infection [9]. Although biofilms are not a direct cause of death, they do cause nosocomial disorders that disrupt the balance of morbidity and mortality in humans [12].

The determination of MBIC (minimum biofilm inhibitory concentration) values is essential in biofilm research. This is because the MBIC value can be used to determine the lowest concentration of antimicrobial agent, which indicates the inhibitory value of the initial biofilm formation. Although this value does not depend on the average number of living cells in the biofilm, the determination of this value becomes a determinant or guide in the treatment of biofilm infections because MBIC is a time-efficient and accurate research method as an antimicrobial agent for biofilm testing [47].

In addition, the MBRC value is determined by the MTT reduction test [40], where the MTT reduction test (MTT assay) is a test that can detect the presence of antibiofilm activity of a compound by reducing MTT saltsin living cells (biofilms) to insoluble formazan [48]. The MTT test assesses microbial viability in biofilms formed in vitro. In determining the tendency of microbes to form biofilms in complex media, the MTT test quantifies formazan from the reduction of tetrazolium salts by the electron transport system [49–52].

Based on the research results obtained, the highest concentration of the C-10 massoia lactone compound extract (1%) gave an inhibitory activity of 80.23% ± 0.01 against *C. tropicalis*. Meanwhile, the lowest concentration (0.125%) showed an inhibitory activity of 70.21% ± 0.01. Although the inhibition results of this middle phase biofilm were not more significant compared to the positive control, which had an inhibitory activity of 85.11% ± 0.01, the C-10 massoia lactone compound was able to inhibit more than 50% (MBIC_50_) of the biofilm formation, even at the lowest concentration of 0.125%.

Based on the research results, the compound C-10 massoia lactone has the potential as an antibiofilm against *C. tropicalis* up to the maturation phase. The compound C-10 massoia lactone at 1% concentration was able to inhibit 74.23% ± 0.01. Although this value is not as high as the drug control, i.e., 78.30% ± 0.01, it is almost equal to the inhibition given by the compound C-10 massoia lactone at a concentration of 1%. Furthermore, even at the lowest concentration, 0.125%, the C-10 massoia lactone extract was still able to inhibit the formation of *C. tropicalis* biofilms by more than 50% (MBIC_50_) with an inhibition of 78.30% ± 0.01 (Table 2).

In previous studies, the C-10 massoia lactone compound was found to be an antibiofilm agent for monomicrobial and polymicrobial organisms. The C-10 massoia lactone compound can inhibit the formation of biofilms of *Pseudomonas aeruginosa*, *Candida albicans*, and *Staphylococcus aureus* [34], polymicrobial biofilms of *Actinomyces viscosus*, *Streptococcus mutants*, *Streptococcus sanguinis*, and *Lactobacillus acidophilus* [35], and polymicrobial biofilms of *Candida albicans*, *Escherichia coli*, and *Pseudomonas aeruginosa* [43]. Massoia oil nanoemulsion preparation (C-10 massoia lactone) provides a robust inhibitory activity on *Pseudomonas aeruginosa* and *Staphylococcus aureus* biofilms compared to other essential oils [53]. In addition, other studies have shown that C-10 massoia lactone may be a potential antibiotic candidate to inhibit biofilm growth, as this compound can disrupt microbial membranes in bacterial and biofilm growth [54]. It is known that the C-10 massoia lactone compound works by disrupting the polysaccharide and lipid matrices in biofilms to disrupt the biofilm formation in microbes [55].

**Figure 2:**
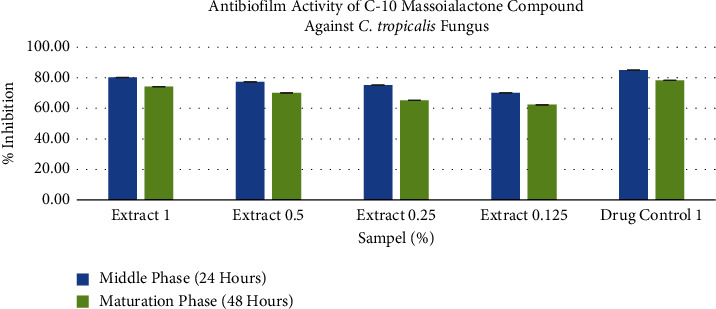
Antibiofilm activity of C-10 massoi lactone against *C. tropicalis* in the middle phase (24 hours) and maturation phase (48 hours).

In the research results obtained (Figure 2), biofilm inhibition was more significant in the middle phase (24 hours) than in the maturation phase (48 hours). This is influenced by the biofilm formation process, where the EPS (extracellular polymeric substances) matrix in the middle phase is not yet as structured and complex as in the ripening phase. The microbes themselves incorporate EPS due to changes in growth rates and gene transcription in planktonic cells or free cells where EPS functions to envelop/enshroud/protect the colonies inside [6, 7, 56].

This statement is also in line with the opinion of Donlan and Costerton [6], Purbowati et al. [10], Kannappan et al. [57], and Hamzah et al. [1], where there will be a decrease in the inhibitory activity of biofilms in the maturation phase (48 hours) compared to the intermediate phase (24 hours) because biofilm growth in the maturation phase has a more complex and structured biofilm defence system so that biofilms in this phase have formed a biofilm defence system. The maturation phase biofilm defense is stronger because the biofilm cells continue to develop for several hours and have a longer lifespan compared to the midphase biofilm.

In addition, Homenta [58], Achinas et al. [59], and Muhammad et al. [47] also confirmed this statement in that the structure of the biofilm cells produced in the maturation phase appears thicker and more complex than that in the intermediate phase. This is because the mucus layer produced by the biofilm is dense and adheres strongly to the wells. This makes it difficult for antibiotics and compounds to penetrate the EPS biofilm layer. EPS on biofilm inhibits the mass transport of antibiotics through biofilm formation. According to Hamzah et al. [60], the biofilm matrix in fungi also acts as the main barrier protecting biofilm cells from attack by antifungal drugs and the body's immune system.

According to Rolli et al. [61], the massoia lactone compound in the masoyi plant is one of the main compounds that can inhibit biofilm formation compared to other compounds.

The results of statistical analysis using Bonferroni post hoc are used to determine which concentration has a difference. The results of the data obtained (Figure 2) show that there are differences or significant differences between each concentration, namely, 1, 0.5, 0.25, and 0.125% w/v, so it is stated (*P* < 0.05). Based on the calculation of percent inhibition between C-10 massoialactone 1% b/v with nystatin drug control (as a positive control), in vitro results showed no significant difference (*P* > 0.05).

### 3.3. Scanning Electron Microscopy (SEM)

**Figure 3:**
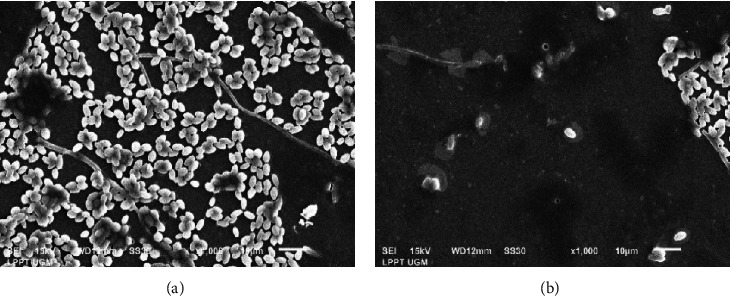
Results of scanning electron microscopy (SEM) of *C. tropicalis* treated with C-10 massoia lactone 0.5% v/v. (a) Before treatment and (b) after treatment.

The compound C-10 massoia lactone at a concentration of 0.5% v/v can cause lysis of *C. tropicalis* biofilm cells, accompanied by a decrease in cell density. Based on Figure 3, SEM results show that the administration of C-10 massoia lactone 0.5% can cause damage to *C. tropicalis* fungi, as the C-10 massoia lactone compound attacks the *C. tropicalis* EPS matrix so that the formation of hyphae is hindered by damage to the fungal cells. There was also a reduction in attachment, density, and lysis of *C. tropicalis* fungal cells.

**Figure 4:**
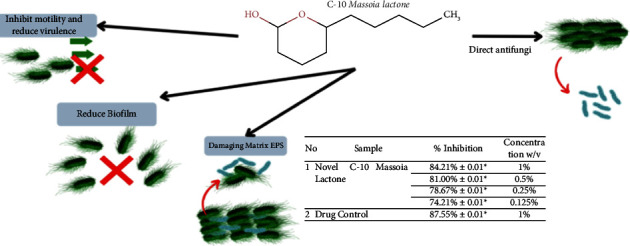
Mechanism of C-10 massoi lactone as an antibiofilm for *C. tropicalis*.

The mechanism of inhibition of the C-10 massoia lactone compound on the growth of intermediate and mature biofilms is thought to be by inhibiting the attachment of microbes to the surface so that biofilm development is disrupted. When biofilm development is disrupted, the structure of the biofilm is affected to enhance antimicrobial defence. The next step is the binding of C-10 massoia lactone compound to bacteria and releasing them from mature biofilms (Figure 4). In addition, the C-10 massoia lactone compound also damages the EPS biofilm so that the communication pathways for cells and nutrients between microbes are cut off, so that the microbes that previously wanted to form biofilms are ultimately unable to form biofilms and cause these microbes to lyse or die due to the loss of nutrients as a component of biofilm formation (Figure 4). Theoretically, the phenolic group is a group that functions in causing antibacterial activity. The compound C-10 massoia lactone has a phenolic group and shows antifungal and antibiofilm activities.

### 3.4. Molecular Docking

To validate the binding of native ligands, the 3D conformation of natural ligands to receptor proteins is searched for, taking into account the coordinates of the centre of mass of the structure and the grid box arrangement of the binding site pocket in units of Å (angstroms) or number of points. The docking results were aligned with the natural ligand conformations, and the measurement results were expressed as root mean square deviation (RMSD) values. The RMSD value for conformational structures that are still acceptable for alignment is less than three, but the most optimal is less than 2 [62]. The closer to 0, the better the alignment value, which means that the output ligand from the docking does not experience much deviation [63]. In our study, we validated using the RMSD value. The following are the RMSD results that we obtained: validity receptor 3HEM (native ligand RMSD: 0.663 (valid, value RMSD <2 Å). The results of the RMSD calculations between the native ligands and the effects of each docking show a value of 0.663 Å, indicating that the method can be continued for molecular docking tests. The best RMSD value is the value that is close to 0. Thus, the first conformation for each ligand compares the conformational value with itself as the best conformation. In addition to looking at the lowest binding energy value, it is also necessary to look at the interaction with the reference compound, as the interaction with the desired residue will affect the role of the combination as an inhibitor.

To propose a molecular explanation for the inhibition of 3HEM enzymes by the compound massoia lactone, the docking score is −6.3. Massoia lactone is primarily associated with hydrogen bonding interactions involving residues TYR 280, TYR 41, PHE 215, and LEU 220. A molecular docking study was performed during the docking procedure, assuming a model in which proteins and ligands are considered rigid and flexible [64]. Estimates of the docking scores, number of interactions, interacting residues, and bond lengths are shown in Table 3.

**Figure 5:**
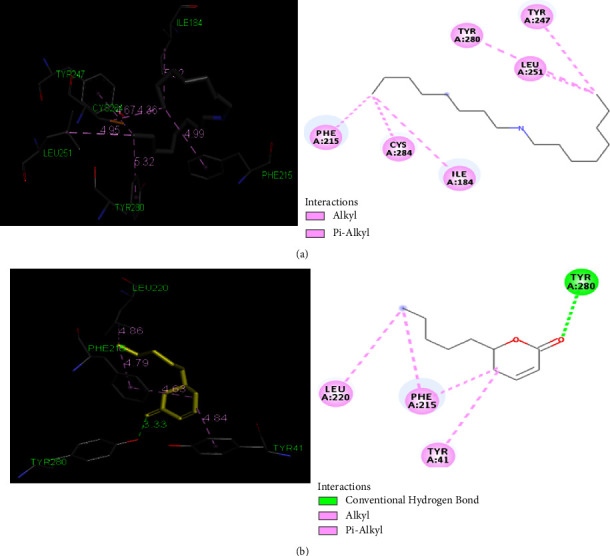
Molecular docking of antifungal (3HEM) with (a) native (b) massoia lactone.

Molecular docking of masoialactone against C. tropicalis can be seen in Figure 5. Proteins are shown as a surface representation. Ligands are shown as a rod representation. A possible explanation could be the limitations of the docking model, namely, how the compounds get to the active site. In all molecular docking experiments, the process starts with the ligand at the active site. “Native” is a natural ligand on the target receptor. The new compound has a strong antibiofilm profile, inhibiting biofilm formation without affecting microbial growth. The interaction shows that the docking massoia lactone showed a significant binding and has the potential as an antifungal agent.

## 4. Conclusion

Based on the research results obtained, the compound C-10 massoia lactone can inhibit the growth of fungi by 84.21% ± 0.01^*∗*^. Meanwhile, the development of *C. tropicalis* biofilm in the intermediate phase was 80.23% ± 0.01^*∗*^, and in the mature phase, it was 74.23% ± 0.01^*∗*^. SEM results showed that C-10 massoia lactone damaged the EPS matrix of *C. tropicalis*, resulting in impaired hyphal formation due to damage to fungal cells, which caused a decrease in attachment, density, and lysis of *C. tropicalis* fungal cells. Based on molecular docking tests, C-10 massoia lactone could inhibit biofilm formation without affecting microbial growth while docking C-10 massoia lactone showed a significant binding and has the potential as an antibacterial agent. In conclusion, the C-10 massoia lactone compound has the potential to be an antibiofilm agent against *C. tropicalis*, so it can become a new antibiofilm agent.

## Figures and Tables

**Table 1:** Percentage of antifungal inhibition of C-10 massoia lactone against *C. tropicalis*.

No.	Sample	% inhibition	Concentration w/v (%)
1	Novel C-10 massoia lactone	84.21% ± 0.01^*∗*^	1
		81.00% ± 0.01^*∗*^	0.5
		78.67% ± 0.01^*∗*^	0.25
		74.21% ± 0.01^*∗*^	0.125

2	Drug control	87.55% ± 0.01^*∗*^	1

**Table 2:** Percentage of antibiofilm inhibition of the C-10 massoia lactone compound against *C. tropicalis* in the middle phase (24 hours) and maturation phase (48 hours).

No.	Sample	% inhibition		Concentration w/v (%)
		Middle phase (24 hours)	Maturation phase (48 hours)	
1	Novel C-10 massoia lactone	80.23% ± 0.01^*∗*^	74.23% ± 0.01^*∗*^	1
		77.36% ± 0.01^*∗*^	70.18% ± 0.01^*∗*^	0.5
		75.21% ± 0.01^*∗*^	65.23% ± 0.01^*∗*^	0.25
		70.21% ± 0.01^*∗*^	62.33% ± 0.01^*∗*^	0.125

2	Drug control	85.11% ± 0.01^*∗*^	78.30% ± 0.01^*∗*^	1

**Table 3:** Docking score, number of interactions, interacting residues, and bond length from the ligand massoia lactone and native to 3HEM target.

(3HEM)				
Ligand	Docking score	Number of interactions	Residual interactions	Bond length (Å)
Native	−5.8	6	TYR 247	4.67
			TYR 280	5.32
			LEU 251	4.95
			PHE 215	4.99
			CYS 284	4.36
			ILE 184	5.32

Massoia lactone	−6.3	4	TYR 280	3.33
			TYR 41	4.84
			PHE 215	4.79
				4.63
			LEU 220	4.86

## Data Availability

The dataset supporting the study's findings is available from the corresponding author upon request.

## References

[1] Hamzah H., Hertiani T., Pratiwi S. U. T., Nuryastuti T. (2020). Inhibitory activity and degradation of curcumin as anti-biofilm polymicrobial on catheters. *International Journal of Research in Pharmacy and Science*.

[2] Ciofu O., Moser C., Jensen P. O., Høiby N. (2022). Tolerance and resistance of microbial biofilms. *Nature Reviews Microbiology*.

[3] Rather M. A., Gupta K., Mandal M. (2021). Microbial biofilm: formation, architecture, antibiotic resistance, and control strategies. *Brazilian Journal of Microbiology*.

[4] Dincer S., Masume Uslu F., Delik A. (2020). Antibiotic resistance in biofilm [internet]. *Bacterial Biofilms*.

[5] Nuryastusi T., Merry M. S., Kholidah S. N., Hertiani T., Asdie R. H., Mustofa G. (2023). *Ulkus Diabetikum: Resistensi Antibiotik & Bakteri Pembentuk Biofilm*.

[6] Donlan R. M., Costerton J. W. (2002). Biofilms: survival mechanisms of clinically relevant microorganisms. *Clinical Microbiology Reviews*.

[7] Choudhary P., Singh S., Agarwal V. (2020). *Microbial Biofilms Princy*.

[8] Anjarwati D. U., Hestiyani R. A. N., Asnani A. (2023). *Staphylococcus Epidermidis Penghasil Biofilm: Resistensi Antibiotik Dan Metode Deteksi Yang Visible*.

[9] Soleha T. U. (2015). Uji kepekaan terhadap antibiotik. *Juke Unila*.

[10] Purbowati R. (2016). Hubungan biofilm dengan infeksi: implikasi pada kesehatan masyarakat dan strategi mengontrolnya. *Jurnal Ilmiah Kedokteran Wijaya Kusuma*.

[11] Muntasir Abdulkadir W. S., Harun A. I., Tenda P. E. (2021). *Antibiotik Dan Resistensi Antibiotik*.

[12] Nasution L. H. (2012). Infeksi nosokomial. *Multiple Disabilities and Vision Impairment (MDVI)*.

[13] Nicolle L. E. (2014). Catheter associated urinary tract infections. *Antimicrobial Resistance and Infection Control*.

[14] Römling U., Kjelleberg S., Normark S. (2014). Microbial biofilm formation: a need to act. *Journal of Internal Medicine*.

[15] Furtuna D. K., Debora K., Wasito E. B. (2018). Antimicrobial susceptibility and the pattern of a biofilm-forming pair of organisms from patients treated in intensive care units in Dr. Soetomo General Hospital, Indonesia. *Bali Medical Journal*.

[16] Cortés M. E., Bonilla J. C., Sinisterra R. D. (2011). Biofilm formation, control and novel strategies for eradication. *Science Against Microbial Pathogens: Communicating Current Research and Technological Advances*.

[17] Azami M., Jaafari Z., Masoumi M. (2019). The etiology and prevalence of urinary tract infection and asymptomatic bacteriuria in pregnant women in Iran: a systematic review and Meta-analysis. *BMC Urology*.

[18] Mouton J. W., Vinks A. A. (2005). Pharmacokinetic/pharmacodynamic modelling of antibacterials in vitro and in vivo using bacterial growth and kill kinetics: the minimum inhibitory concentration versus stationary concentration. *Clinical Pharmacokinetics*.

[19] Mulyadi M., Wuryanti W., Sarjono P. R. (2017). Konsentrasi hambat minimum (KHM) kadar sampel alang-alang (*Imperata cylindrica*) dalam etanol melalui metode difusi cakram. *Jurnal Kimia Sains dan Aplikasi*.

[20] Parvekar P., Palaskar J., Metgud S., Maria R., Dutta S. (2020). The minimum inhibitory concentration (MIC) and minimum bactericidal concentration (MBC) of silver nanoparticles against *Staphylococcus aureus*. *Biomaterial Investigations in Dentistry*.

[21] Tarigan B. M. C. B., Lelyana S., Sugiaman V. K. (2021). Kadar hambat minimum dan kadar bunuh minimum ekstrak etanol daun oregano terhadap pertumbuhan Candida albicans. *Jurnal Ilmiah dan Teknologi Kedokteran Gigi FKG UPDM (B)*.

[22] Fauzia D. (2017). Strategi optimasi penggunaan antibiotik. *Jurnal Ilmu Kedokteran*.

[23] Mancuso G., Midiri A., Gerace E., Biondo C. (2021). Bacterial antibiotic resistance: the most critical pathogens. *Pathogens*.

[24] Wang C. H., Hsieh Y. H., Powers Z. M., Kao C. Y. (2020). Defeating antibiotic-resistant bacteria: exploring alternative therapies for a post-antibiotic era. *International Journal of Molecular Sciences*.

[25] Altemimi A., Lakhssassi N., Baharlouei A., Watson D. G., Lightfoot D. A. (2017). Phytochemicals: extraction, isolation, and identification of bioactive compounds from plant extracts. *Plants*.

[26] Puspita D., Wulandari T. S. (2020). Analisis senyawa bioaktif pada daun kemangi imbo (Pycnarrhena cauliflora) yang digunakan sebagai penyedap alami. *Jurnal Teknologi Pangan dan Gizi*.

[27] Rossiter S. E., Fletcher M. H., Wuest W. M. (2017). Natural products as platforms to overcome antibiotic resistance. *Chemical Reviews*.

[28] Álvarez-Martínez F. J., Barrajón-Catalán E., Micol V. (2020). Tackling antibiotic resistance with compounds of natural origin: a comprehensive review. *Biomedicines*.

[29] Hamzah H., Pratiwi S. U. T., Hertiani T. (2022). Efficacy of C-10 massoialactone against-multispecies microbial biofilm. *Biointerface Research in Applied Chemistry*.

[30] Rollando R., Prasetyo Y. S. A., Sitepu R. (2019). Uji antimikroba minyak atsiri masoyi (Massoia aromatica) terhadap bakteri Streptococcus mutans. *Majalah Farmasi dan Farmakologi*.

[31] Zhang M., Gao Z. C., Chi Z. (2022). Massoia lactone displays strong antifungal property against many crop pathogens and its potential application. *Microbial Ecology*.

[32] Ningsih S. C., Hamzah H. (2022). Penelusuran pemanfaatan dan bioaktivitas tanaman masoyi (Cryptocarya massoy): review. *Jurnal Farmagazine*.

[33] Hertiani T., Yuswanto A., Utami Tunjung Pratiwi S., Muthma’innah Mashar H. (2018). Effect of massoia (massoia aromatica becc.) bark on the phagocytic activity of wistar rat macrophages. *Scientia Pharmaceutica*.

[34] Pratiwi S. U. T., Hertiani T., Idroes R., Lagendijk E. L., de Weert S., Hondel C. V. D. (2016). Quorum quenching and biofilm-degrading activity of massoia oil against Candida albicans and *Pseudomonas aeruginosa*. *Planta Medica*.

[35] Utami D. T., Tunjung Pratiwi S. U., Spaink H. P., Haniastuti T., Hertiani T. (2021). Antibiofilm effect of C-10 massoia lactone toward polymicrobial oral biofilms. *Journal of Advanced Pharmaceutical Technology and Research*.

[36] da Costa J. S., Barroso A. S., Mourão R. H. V., da Silva J. K. R., Maia J. G. S., Figueiredo P. L. B. (2020). Seasonal and antioxidant evaluation of essential oil from Eugenia uniflora L., curzerene-rich, thermally produced in situ. *Biomolecules*.

[37] Barbara D., Alexander M. D., Mhs C. L. S. I. (2017). *Reference Method for Broth Dilution Antifungal Susceptibility Testing of Yeasts*.

[38] Pierce C. G., Uppuluri P., Tummala S., Lopez-Ribot J. L. (2010). A 96 well microtiter plate-based method for monitoring formation and antifungal susceptibility testing of Candida albicans biofilms. *Journal of Visualized Experiments: JoVE*.

[39] Hamzah H., Tunjung Pratiwi S. U., Hertiani T. (2018). Efficacy of thymol and eugenol against polymicrobial biofilm. *Indonesian Journal of Pharmacy*.

[40] Nuryastuti T., Setiawati S., Ngatidjan N. (2018). Antibiofilm activity of (1)-N-2-methoxybenzyl-1,10-phenanthrolinium bromide against Candida albicans. *Journal de Mycologie Médicale*.

[41] Ali I., Khan F. G., Suri K. A. (2010). In vitro antifungal activity of hydroxychavicol isolated from Piper betle L. *Clinical Microbiology and Antimicrobials*.

[42] Pratiwi S. U. T., Hertiani T. (2017). Efficacy of massoia oil in combination with some Indonesian medicinal plants oils as anti-biofilm agent towards Candida albicans. *International Journal of Pharmaceutical Sciences and Research*.

[43] Relucenti M., Familiari G., Donfrancesco O. (2021). Microscopy methods for biofilm imaging: focus on SEM and VP-SEM pros and cons. *Biology*.

[44] Syamsul E. S., Umar S., Wahyuni F. S., Martien R., Hamidi D. (2022). Antiaging activity, in silico modeling and molecular docking from sonneratia caseolaris. *Open Access Macedonian Journal of Medical Sciences*.

[45] Vishnu V. R., Renjith R. S., Mukherjee A., Anil S. R., Sreekumar J., Jyothi A. N. (2019). Comparative study on the chemical structure and in vitro antiproliferative activity of anthocyanins in purple root tubers and leaves of sweet potato (Ipomoea batatas). *Journal of Agricultural and Food Chemistry*.

[46] Zoccolotti J. D. O., Cavalheiro A. J., Tasso C. O., Ribas B. R., Ferrisse T. M., Jorge J. H. (2021). Antimicrobial efficacy and biocompatibility of extracts from Cryptocarya species. *PLoS One*.

[47] Muhammad M. H., Idris A. L., Fan X. (2020). Beyond risk: bacterial biofilms and their regulating approaches. *Frontiers in Microbiology*.

[48] Siregar F., Hadijono B. S. (2000). Uji sitotoksisitas dengan esei MTT. *Jurnal Kedokteran Gigi Universitas Indonesia*.

[49] Kitagawa N., Shiota S., Shibata Y., Takeshita T., Yamashita Y. (2011). Characterization of MbrC involved in bacitracin resistance in Streptococcus mutans. *FEMS Microbiology Letters*.

[50] Trafny E. A., Lewandowski R., Zawistowska-Marciniak I., Stępińska M. (2013). Use of MTT assay for determination of the biofilm formation capacity of microorganisms in metalworking fluids. *World Journal of Microbiology and Biotechnology*.

[51] Feng J., Wang T., Zhang S., Shi W., Zhang Y. (2014). An optimized SYBR Green I/PI assay for rapid viability assessment and antibiotic susceptibility testing for Borrelia burgdorferi. *PLoS One*.

[52] de Oliveira Pinto Ribeiro A., Carolina da Silva A., de Camargo Ribeiro F. (2022). Biofilm formation and cell viability on monolithic zirconia with silver-doped sodalime glass. *Journal of the Mechanical Behavior of Biomedical Materials*.

[53] Hertiani T., Pratiwi S. U. T., Yuswanto A., Permanasari P. (2016). Potency of massoia bark in combating immunosuppressed-related infection. *Pharmacognosy Magazine*.

[54] Yasir M., Willcox M. D., Dutta D. (2018). Action of antimicrobial peptides against bacterial biofilms. *Materials*.

[55] Cowan M. M. (1999). Plant products as antimicrobial agents. *Clinical Microbiology Reviews*.

[56] Gunardi W. D. (2014). Peranan biofilm dalam kaitannya dengan penyakit infeksi. *Jurnal Kedokteran Meditek*.

[57] Kannappan A., Sivaranjani M., Srinivasan R., Rathna J., Pandian S. K., Ravi A. V. (2017). Inhibitory efficacy of geraniol on biofilm formation and development of adaptive resistance in Staphylococcus epidermidis RP62A. *Journal of Medical Microbiology*.

[58] Homenta H. (2016). Infeksi biofilm bakterial. *Jurnal E-Biomedik*.

[59] Achinas S., Charalampogiannis N., Euverink G. J. W. (2019). Brief recap for bacteria adhesion and biofilms. *Applied Siences*.

[60] Hamzah H., Rasdianah N., Nurwijayanto A., Nandini E. (2021). Aktivitas ekstrak etanol daun calincing terhadap biofilm Candida albicans. *Jurnal Farmasetis*.

[61] Rolli E., Marieschi M., Maietti S. (2016). Phytotoxic effects and phytochemical fingerprinting of hydrodistilled oil, enriched fractions, and isolated compounds obtained from Cryptocarya massoy (oken) kosterm. Bark. *Chemistry and Biodiversity*.

[62] Carugo O. (2003). How root-mean-square distance (r.m.s.d.) values depend on the resolution of protein structures that are compared. *Journal of Applied Crystallography*.

[63] Imbernón B., Serrano A., Bueno-Crespo A., Abellán J. L., Pérez-Sánchez H., Cecilia J. M. (2020). Metadock 2: a high-throughput parallel metaheuristic scheme for molecular docking. *Bioinformatics*.

[64] Zeng H. J., Yang R., You J., Qu L. B., Sun Y. J. (2016). Spectroscopic and docking studies on the binding of liquiritigenin with hyaluronidase for antiallergic mechanism. *Scientific*.

